# Chemical characterization of extra layers at the interfaces in MOCVD InGaP/GaAs junctions by electron beam methods

**DOI:** 10.1186/1556-276X-6-194

**Published:** 2011-03-03

**Authors:** Cesare Frigeri, Alexey Aleksandrovich Shakhmin, Dmitry Anatolievich Vinokurov, Maria Vladimirovna Zamoryanskaya

**Affiliations:** 1 CNR-IMEM Institute, Parco Area delle Scienze 37/A, 43010 Parma, Italy; 2 Ioffe Physical Technical Institute, 194021 Polytekhnicheskaya 26, Saint-Petersburg, Russia

## Abstract

Electron beam methods, such as cathodoluminescence (CL) that is based on an electron-probe microanalyser, and (200) dark field and high angle annular dark field (HAADF) in a scanning transmission electron microscope, are used to study the deterioration of interfaces in InGaP/GaAs system with the GaAs QW on top of InGaP. A CL emission peak different from that of the QW was detected. By using HAADF, it is found that the GaAs QW does not exist any longer, being replaced by extra interlayer(s) that are different from GaAs and InGaP because of atomic rearrangements at the interface. The nature and composition of the interlayer(s) are determined by HAADF. Such changes of the nominal GaAs QW can account for the emission observed by CL.

## Introduction

Several devices, such as HBTs, HEMTs, solar cells and LEDs, are currently based on InGaP/GaAs heterojunction because of its superior properties with respect to AlGaAs [1-4]. The InGaP/GaAs system, especially if it is grown by metal organic vapour phase deposition (MOCVD), has, however, the drawback that the interfaces between InGaP and GaAs are deteriorated, as shown by photoluminescence, X-ray diffraction and transmission electron microscopy (TEM), because there is no common group V element across the interface [5]. This mostly affects the inverted GaAs-on-InGaP interface where an unwanted extra interlayer forms, which recombines the minority carriers more efficiently than the GaAs quantum well [5-10]. The normal InGaP-on-GaAs interface is always good, but this is not sufficient to guarantee reliable device performance. The deterioration of the inverted GaAs-on-InGaP interface has been seen to occur in practically every MOCVD InGaP/GaAs heterostructure containing such an interface, to a more or less great extent depending on the growth conditions [5-10]. It could sometimes be avoided by the use of growth interruption between the layers [6], the growth on top of InGaP of a thin (1 nm) intentional interfacial layer of GaP [5,7,9] or GaAlAs [8], or the application of a preflow of trimethylgallium on the InGaP surface before switching on the AsH_3 _flow [11].

A recent contribution to this field was based on cathodoluminescence (CL) measurements [12,13]. The difference between the two interfaces was confirmed by comparing two InGaP/GaAs systems containing a GaAs QW and either one of the two interfaces [12,13]. One sample had the layer sequence GaAs substrate/GaAs buffer/AlGaAs/GaAs/InGaP with the normal interface. It showed the expected GaAs QW emission (1.56 eV at 77 K). The other sample had the sequence GaAs substrate/GaAs buffer/InGaP/GaAs/AlGaAs with the inverted GaAs-on-InGaP interface. This sample did not exhibit the expected QW emission. On the contrary, a CL peak was seen at 1.48 eV, which suggested that the GaAs QW was absent, having been replaced by a transition layer of InGaAsP with mixed composition [12,13]. The aim of this study is to check by TEM whether the CL results can be related to structural modifications of the GaAs QW, such as the presence of an interlayer of the type described earlier. An additional objective is to determine the composition of any extra layer that could have been formed by using the innovative chemically sensitive high angle annular dark field (HAADF) method in a scanning TEM thanks to its square dependence on the atomic number.

## Experiment

The InGaP/GaAs structures were grown by MOCVD at 973 K using an Emcore GS3100 reactor, and they had the following layer sequence: (100) GaAs substrate/GaAs buffer (180 nm)/InGaP (130 nm)/GaAs QW (10 nm)/AlGaAs (370 nm) cap. The expected layer thickness is given in brackets. Both CL and TEM gave 160 nm for InGaP, 360 nm for AlGaAs and 10 ± 1 nm for QW. They were analysed by spectroscopic CL and TEM. CL was done at temperatures of 300 and 77 K in an electron-probe microanalyser Camebax supplied with the CL system. TEM observations were done in an FEG 2200FS JEOL instrument on <011> cross-sectional specimens prepared by the standard sandwich procedure and finally thinned with Ar ion bombardment. The (200) dark field (DF) mode and the HAADF method in association with the scanning operation of the TEM (STEM) were used for detection of interface modifications and composition.

## Results and discussion

**Figure 1:**
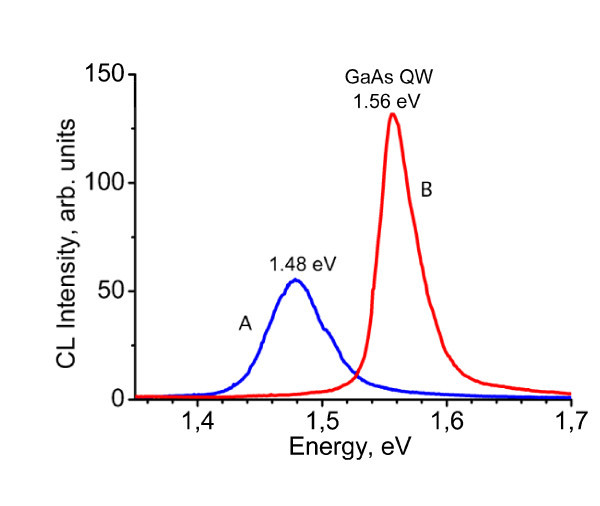
**CL spectrum A in the near-IR region of the investigated sample compared with that (B) of a sample exhibiting the expected GaAs QW emission**. CL at 77 K with 5 keV beam energy.

Different electron beam energies were used to check the in-depth distribution of the layers. In the CL spectra at 77 K, bands corresponding to AlGaAs layer at 1.89 eV and InGaP layer at 1.94 eV were detected at the expected depth, indicating a composition of Al_0.26_Ga_0.74_As and In_0.51_Ga_0.49_P, respectively [12,13]. However, the emission from the GaAs QW was not detected; only a wide luminescence band at 1.48 eV, which could rather correspond to bulk GaAs, was observed as shown in the CL spectrum in the near-infrared (IR) region of Figure 1, where the CL emission A of the sample studied here is compared with the peak B of the GaAs QW (1.56 eV at 77 K) observed in a similar structure but containing the normal InGaP-on-GaAs interface, i.e. GaAs substrate/GaAs buffer/AlGaAs/GaAs/InGaP [12,13].

**Figure 2:**
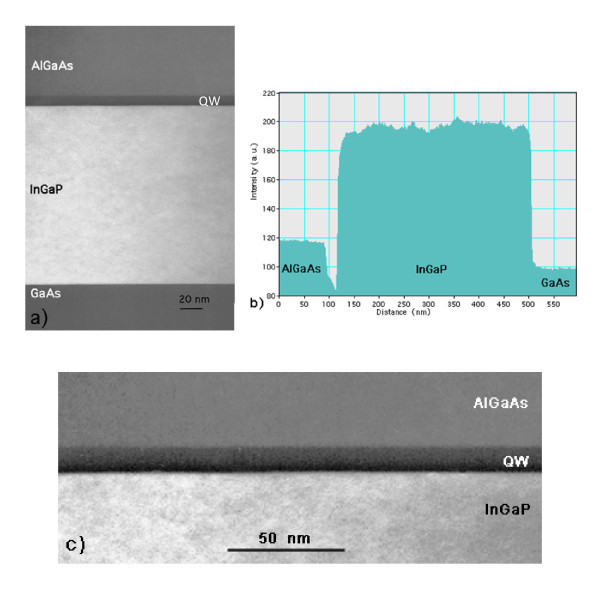
**(a) (200) DF TEM image of the sample and (b) intensity profile across it along the negative growth direction**. In **(a)**, the nominal GaAs QW is the dark stripe between InGaP and AlGaAs and corresponds to the downward peak between InGaP and AlGaAs in **(b)**. The profile **(b) **clearly shows that it exhibits a contrast darker than the GaAs substrate/buffer (at the right-hand side). **(c) **High-magnification (200) DF image of the GaAs QW. The image has been treated with Adobe Photoshop to improve the visibility of the extra layer in proximity of the GaAs-on-InGaP interface.

To check the reason for such anomalous emission, TEM (200) DF and STEM-HAADF were applied. Figure 2 shows the (200) DF TEM image of the sample. The nominal GaAs QW layer is the dark stripe between InGaP and AlGaAs. It exhibits a contrast darker than the GaAs substrate/buffer as seen in Figure 2b. This suggests that this layer is not GaAs. Figure 2c shows the high-magnification image of the nominal QW showing two different contrasts inside it in agreement with the profile of Figure 2b, confirming that the nominal QW is made up of two sublayers, as could also be concluded from Figure 2b. As the images were acquired in thin areas of the TEM specimen, the kinematical approximation is used, according to which the (200) DF intensity *I*_200 _is proportional to $F_{200}^{2}$, with *F*_200 _as the structure factor of the (200) diffraction that depends on the atomic scattering factors f of the elements in the III-V compound as it is *F*_200 _= 4(*f*_III _- *f*_V_) [14-16]. To evaluate composition, the DF contrast function *C*_200_, which is defined as the ratio between the (200) DF intensity diffracted by a given layer of general form *A_x_B*_1-*x*_*C_y_D*_1-*y *_and that diffracted by GaAs, is used. An alloy looks darker than GaAs when *C*_200 _is <1. *C*_200 _depends on the square of the composition as does $F_{200}^{2}$[14-16] because *f*_III _and *f*_V _have to be introduced in proportion to the relative composition of the element they refer to.

**Figure 3:**
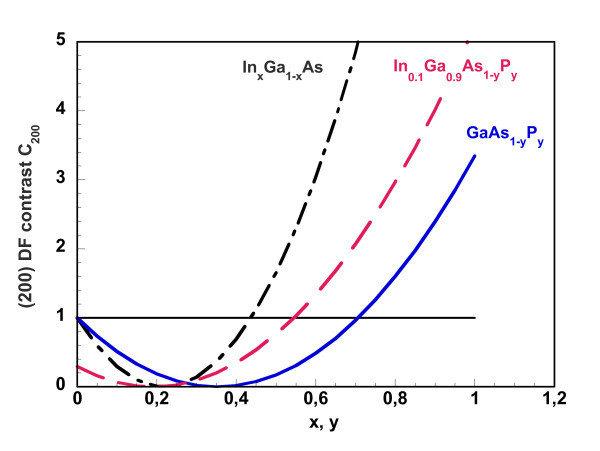
**Calculated (200) DF contrast function *C*_200 _for In_*x*_Ga_1-*x*_As (dash and dot line), GaAs_1-*y*_P_*y*_(solid line) and In_*x*_Ga_1-*x*_As_1-*y*_P_*y*_with *x *= 0.1 (dash line) (see text)**.

Computed plots of *C*_200 _for In_*x*_Ga_1-*x*_As and GaAs_1-*y*_P_*y *_are given in Figure 3. These plots show that these two alloys look darker than GaAs for *x *< 0.437 and *y *< 0.707, respectively. In_*x*_Ga_1-*x*_As_1-*y*_P_*y *_is also darker than GaAs for *x *< 0.437 and *y *< 0.707 as is seen by similar plots; by way of example, only the plot for In_*x*_Ga_1-*x*_As_1-*y*_P_*y *_with *x *= 0.1 is shown in Figure 3. No other alloy has *C*_200 _< 1. Though (200) DF can clearly tell which alloy had formed in place of the nominal GaAs QW at the inverted GaAs-on-InGaP interface, no exact estimation of the composition is straightforward because of the square dependence of *C*_200 _on the composition and the indication of just a composition range.

**Figure 4:**
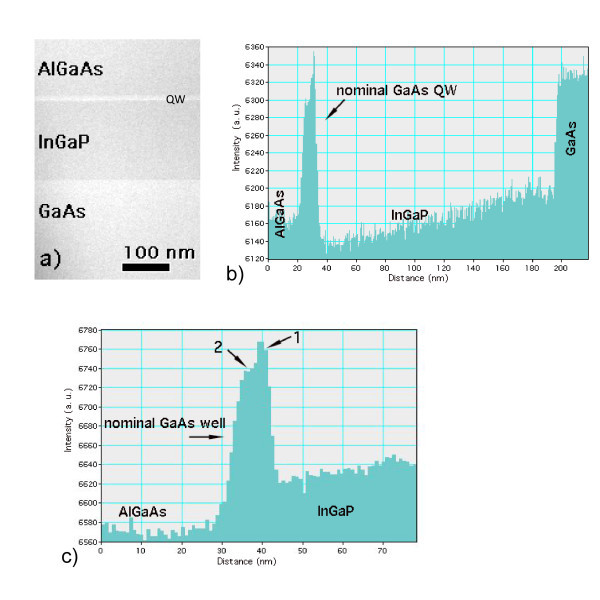
**(a) STEM-HAADF image of the whole structure**. The nominal GaAs QW is the bright stripe between the InGaP and AlGaAs barriers. **(b,c) **HAADF intensity profile across **(a) **and only across the nominal GaAs QW at higher magnification, respectively. Intensity scan along the negative growth direction. In **(c)**, 1 and 2 indicate the two sublayers replacing the nominal GaAs QW (see text).

To evaluate better the composition, the STEM-HAADF method was used. The STEM-HAADF image of the whole structure is given in Figure 4a. The intensity profile of Figure 4b shows that the contrast at the nominal GaAs layer is different from that of the GaAs substrate, confirming the DF results that the nominal QW is no longer made of GaAs. It also shows that the nominal GaAs well is made up of two sublayers, 1 and 2, with appreciable difference in HAADF contrast (Figure 4b,c); sublayer 1 (4 nm thick), which is closer to the GaAs-on-InGaP interface, with a contrast higher than the GaAs substrate, and sublayer 2 (6 nm thick), which is on the side of the AlGaAs barrier, with a lower contrast.

**Figure 5:**
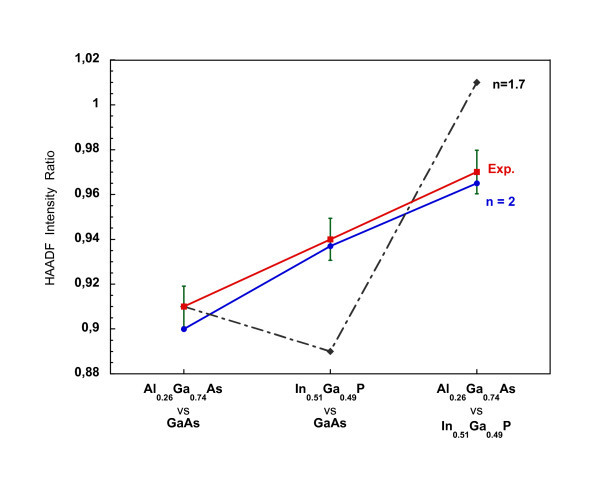
**Choice of the exponent *n***. Calculated HAADF intensity ratios between the three inner standards, taken two by two, for *n *= 1.7 (black dash and dot line, dark lozenges) and *n *= 2 (blue solid line, blue circles) as compared to the relevant experimental ratios (red solid line, red squares). Within experimental errors, exponent *n *= 2 fits very well to the experiment.

The HAADF image is formed by collecting the incoherently scattered electrons at high angles [17,18]. Single atoms scatter incoherently, and the image intensity is the sum of the individual atomic scattering contributions [19]. The higher the atomic number *Z*, the larger the scattering angle is. The HAADF intensity turns out to be proportional to *Z^n, ^*with *n *= 2 [17,18,20], so that a more direct evaluation of the composition is possible. Such dependence could also take other values for the exponent *n*, i.e. 1.7 <*n *< 2 [20]. Here it is assumed that *n *= 2. This choice stems from the fact that only the exponent 2 can fully account for our experimental ratios of the intensities of every couple of layers of known composition in the structures (GaAs substrate/buffer, In_0.51_Ga_0.49_P, Al_0.26_Ga_0.74_As, taken as two by two) as shown in Figure 5, where the calculated HAADF intensity ratios for the two extreme cases of *n *= 1.7 and *n *= 2 are compared with the experimental ratios. The best agreement between the calculated rations and those of the experiment is obtained for *n *= 2.

The composition of the nominal GaAs QW is determined from HAADF pictures by taking the known compositions of the other alloys (GaAs substrate/buffer, In_0.51_Ga_0.49_P, Al_0.26_Ga_0.74_As) and related HAADF intensity values as reference, i.e. as internal standards. The ratios of the experimental intensity of sublayers 1 and 2 to the intensity of all the inner standards are then compared to the calculated values of similar ratios for all the alloys that can be formed by combining together all the elements present at the inverted GaAs-on-InGaP interface assuming the *Z*^2 ^dependence of the intensities. The ratio *R *of the HAADF intensity of a generic sublayer (subl) *A_p_B_q_C_r _*to the one of a generic standard (std) *E_k_F_m_G_n _*is calculated from the equation:

(1) $$R=\frac{I_{\text{HAADF}}^{\text{subl}}(A_{p}B_{q}C_{r})}{I_{\text{HAADF}}^{\text{std}}(E_{k}F_{m}G_{n})}=\frac{pZ_{A}^{2}+qZ_{B}^{2}+rZ_{C}^{2}}{kZ_{E}^{2}+mZ_{F}^{2}+nZ_{G}^{2}}$$

The alloy whose *R *matches the experimental ratio *R*_exp _is the one that a sublayer is made of.

The experimental ratios *R*_exp _of the HAADF intensity of sublayer 1 of the nominal GaAs layer to those of the GaAs substrate, In_0.51_Ga_0.49_P and Al_0.26_Ga_0.74_As, are *R*_exp _= 1.02, *R*_exp _= 1.09 and *R*_exp _= 1.12, respectively (Table 1). For sublayer 2 of the nominal GaAs QW, the same ratios are 0.97, 1.03 and 1.06, respectively (Table 2). The compounds that exhibit ratio *R *of their calculated intensities to GaAs substrate, In_0.51_Ga_0.49_P and Al_0.26_Ga_0.74_As, in the same range as the experimental values given above are only In_*x*_Ga_1-*x*_As, GaAs_1-*y*_P_*y *_and In_*x*_Ga_1-*x*_As_1-*y*_P_*y*_, which are in fairly good qualitative agreement with (200) DF. The other alloys that may be formed at the inverted interface yield (much) different ratios for any possible composition.

**Table 1:** Values of the experimental ratio *R*_exp _of the HAADF intensity *I*_HAADF _of sublayer #1 to those of the three alloys (GaAs substrate, In_0.51_Ga_0.49_P and Al_0.26_Ga_0.74_As) contained in the sample and used as standards.

	$\frac{I_{\text{HAADF}}(\text{Subl}\#1)}{I_{\text{HAADF}}(\text{GaAs})}$	$\frac{I_{\text{HAADF}}(\text{Subl}\#1)}{I_{\text{HAADF}}({\text{In}}_{0.51}{\text{Ga}}_{0.49}\text{P})}$	$\frac{I_{\text{HAADF}}(\text{Subl}\#1)}{I_{\text{HAADF}}({\text{Al}}_{0.26}{\text{Ga}}_{0.74}\text{As})}$
*R*_exp_	1.02	1.09	1.12

**Table 2:** Values of the experimental ratio *R*_exp _of the HAADF intensity *I*_HAADF _of sublayer #2 to those of the three alloys (GaAs substrate, In_0.51_Ga_0.49_P and Al_0.26_Ga_0.74_As) contained in the sample and used as standards

	$\frac{I_{\text{HAADF}}(\text{Subl}\#2)}{I_{\text{HAADF}}(\text{GaAs})}$	$\frac{I_{\text{HAADF}}(\text{Subl}\#2)}{I_{\text{HAADF}}({\text{In}}_{0.51}{\text{Ga}}_{0.49}\text{P})}$	$\frac{I_{\text{HAADF}}(\text{Subl}\#2)}{I_{\text{HAADF}}({\text{Al}}_{0.26}{\text{Ga}}_{0.74}\text{As})}$
*R*_exp_	0.97	1.03	1.06

**Figure 6:**
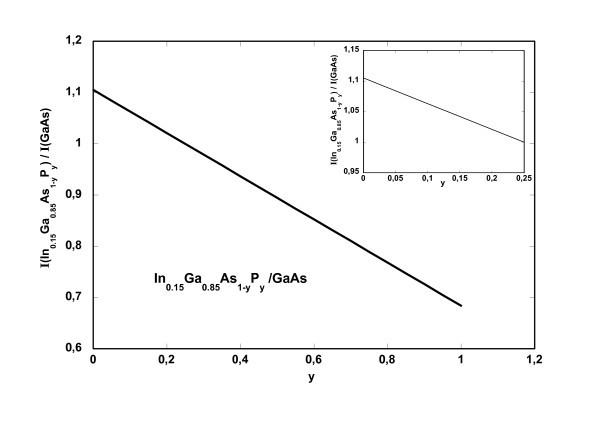
**Plot of the calculated ratio R between the HAADF intensities of In_0.15_Ga_0.85_As_1-*y*_P_y _and GaAs**. Inset is the top left part of the plot.

Figure 6 is a worked-out example of the procedure used to extract information on the nature and composition of sublayers 1 and 2. Figure 6 is the plot of the calculated intensity ratio between In_0.15_Ga_0.85_As_1-*y*_P_*y *_and GaAs. It shows that the experimental value of *R*_exp _= 1.02 for sublayer 1 can be accounted for if the layer is In_0.15_Ga_0.85_As_0.81_P_0.19_. A similar plot for In_*x*_Ga_1-*x*_As to GaAs shows that In_0.03_Ga_0.97_As also fits the experimental result *R*_exp _= 1.02. The same procedure applied using the In_0.51_Ga_0.49_P and Al_0.26_Ga_0.74_As layers as standards leads to the same results for the stoichiometric indices, within 5%. By taking average values, it turns out that the sublayer 1 can be either In_0.15_Ga_0.85_As_0.80_P_0.20 _or In_0.023_Ga_0.977_As. As for sublayer 2 of the nominal GaAs QW, it results in either In_0.05_Ga_0.95_As_0.84_P_0.16 _or GaAs_0.91_P_0.09 _by the same procedure.

**Figure 7:**
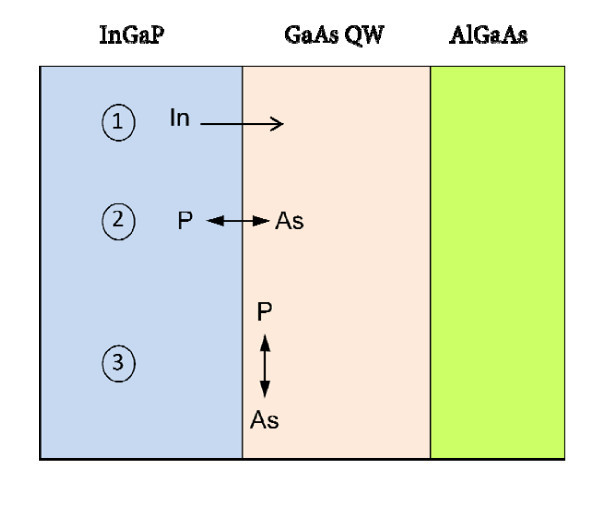
**Sketch of the three possible mechanisms of atom rearrangement at the inverted GaAs-on-InGaP interface**. 1): indium segregation in the growth direction, 2): P/As exchange across the interface, and 3): P/As intermixing in the growing GaAs QW (see text).

The TEM results indicating the formation of InGaAsP at the location of the nominal GaAs QW are in qualitative agreement with an analogous conclusion drawn by CL in refs. [12,13], where a quaternary with the In composition in the 0-0.15 range and the P one a little above zero was proposed. Both the TEM and CL results suggest that at the inverted GaAs-on-InGaP interface there is the formation of an extra quaternary layer of InGaAsP inside the nominal GaAs QW (and partially replacing it), as also suggested in several studies [5,6,8-10,16,21]. The formation of just InGaAs as sublayer 1 might be less likely because it might easily happen that residual P atoms, which remained in the reactor after the PH_3 _flow had been switched off, are incorporated in the first monolayers of the GaAs QW, since Ga prefers to bond to P rather than to As [22], as long as P atoms are available (P/As intermixing mechanism, see later). Moreover, the absence of P in sublayer 1 would contradict its presence in sublayer 2. On the other hand, the sequence inside the nominal GaAs QW such as layer 1 = In_0.15_Ga_0.85_As_0.80_P_0.20 _and layer 2 = In_0.05_Ga_0.95_As_0.84_P_0.16 _or GaAs_0.91_P_0.09 _is congruent. In fact, it matches the reasonable expectation that [In] and [P] decrease by moving away from InGaP, i.e. by going deeper into the nominal GaAs QW, while [Ga] and [As] increase. The stoichiometry of the sublayers 1 and 2 as determined by STEM-HAADF thus indicates a slight In and P enrichment of the nominal GaAs QW, which therefore changes its nature. Three mechanisms can cause such In and P enrichment, namely, In segregation in the growth direction, P/As exchange across the interface and P/As intermixing in proximity of the inverted interface, as discussed in other studies [5,6,8-10,16,21]. The three mechanisms are sketched in Figure 7. Indium surface segregation has been shown for other In-containing systems such as InGaAs/GaAs [23,24]. For the InGaP/GaAs system, the action of In segregation has been proven by experiments, showing that the growth of a thin GaP layer on the top of InGaP, before GaAs is grown, is effective in preventing the formation of the quaternary interlayer because In segregates into the interposed GaP layer and cannot reach the GaAs [5]. In segregates into the growing GaAs layer as soon as the latter starts to grow. In segregation is a kinetically driven process and depends strongly on the growth temperature [5]. It may occur within the first few monolayers of the layer grown next [5,23,24]. P/As exchange across the interface should be excluded according to our results. In fact, this mechanism would entail the incorporation of As in the bottom InGaP with the formation of some InGaAsP alloy inside the nominal InGaP layer, with the consequent broadening of the interface towards both the nominal InGaP and GaAs layers. These detailed investigations by chemically sensitive methods in a TEM right of the inverted interface do not confirm such symmetrical broadening and allow excluding the P/As exchange mechanism. The interface broadening towards only the top GaAs layer was observed by TEM also in other MOVPE-grown InGaP/GaAs samples [16]. P/As intermixing occurs at the beginning of GaAs growth after the growth of InGaP has finished. It consists in the fact that when the Ga and As fluxes are switched on to grow GaAs, some of the incoming Ga atoms bond to residual P atoms that are still remaining in the MOCVD chamber in contact with the sample surface after the PH_3 _flux has been switched off. This is because the chemical bond strength of Ga-P is greater than that of Ga-As [22], which results in As substitution by P [9,22]. Such intermixing is limited to the first monolayers of the growing nominal GaAs because the residual P atoms vanish out very quickly as no PH_3 _flux is active. As for In segregation, P/As intermixing also depends on the substrate temperature which affects, e.g., the diffusion length of the P, As and Ga atoms on the growing surface. It also depends on the gas fluxes, on the application or non-application of a PH_3_-purging procedure or growth interruption [6]. Although the formation of an extra layer at the inverted interface during growth has been reported in a majority of the literature [5-10, 21 and references therein], its composition was seen to vary depending on the growth conditions used, as summarized above. In fact, it has been seen by photoluminescence that the emission associated with the extra layer spans quite a wide range, i.e. from 862 to 914 nm [5-10,21]. A majority of the published articles concluded that the extra layer is InGaAsP albeit with different compositions. Our results agree with this hypothesis. They also show that a finer structure may exist in the modified nominal GaAs QW, i.e. the presence of two sublayers: one more In- and P-rich layer closer to the undergrown InGaP layer and a second one that is less In and P rich farther from it. This structure is certainly due to the expected reduction of P/As intermixing and In segregation as the distance from the inverted interface increases.

## Conclusions

The deterioration of the structure of the GaAs QW in an InGaP/GaAs/AlGaAs heterostructure grown by MOCVD has been studied by CL and (S)TEM. The chemically sensitive (200) DF and HAADF methods of (S)TEM helped us to establish that the nominal GaAs QW has changed its structure, being replaced by two sublayers made of InGaAsP with different compositions. The sublayer closer to the inverted GaAs-on-InGaP interface is more In and P rich than the one on the side of the AlGaAs-on-GaAs interface. The composition of the extra layer of InGaAsP closer to the inverted GaAs-on-InGaP interface, as determined by STEM-HAADF, reasonably accounts for the anomalous emission measured by CL. The formation of the extra layers during growth was ascribed to the rearrangement of the atoms available at the inverted GaAs-on-InGaP interface caused by In segregation in the growth direction and P/As intermixing during the early stages of the GaAs QW growth.

## Abbreviations

CL: cathodoluminescence; HAADF: high-angle annular dark field; MOCVD: metal organic vapour phase deposition; STEM: scanning TEM; TEM: transmission electron microscopy.

## Competing interests

The authors declare that they have no competing interests.

## Authors' contributions

CF made substantial intellectual contributions to the study. Carried out the TEM-HAADF, made the results interpretation and wrote the paper. AAS performed the measurement and interpretation of the cathodoluminescence results and revised the manuscript. DAV made the design and MOCVD growth of the heterostructures. MVZ supervised the work, participated in discussion of the results and in revising the manuscript.
